# Joint associations of multiple leisure-time sedentary behaviours and physical activity with obesity in Australian adults

**DOI:** 10.1186/1479-5868-5-35

**Published:** 2008-07-01

**Authors:** Takemi Sugiyama, Genevieve N Healy, David W Dunstan, Jo Salmon, Neville Owen

**Affiliations:** 1 Cancer Prevention Research Centre, School of Population Health, The University of Queensland, Brisbane, Queensland, Australia; 2 Baker IDI Heart and Diabetes Institute, Melbourne, Victoria, Australia; 3 Centre for Physical Activity and Nutrition Research, School of Exercise and Nutrition Sciences, Deakin University, Melbourne, Victoria, Australia

## Abstract

**Background:**

Television viewing and physical inactivity are independently associated with risk of obesity. However, how the combination of multiple leisure-time sedentary behaviours (LTSB) and physical activity (LTPA) may contribute to the risk of obesity is not well understood. We examined the joint associations of multiple sedentary behaviours and physical activity with the odds of being overweight or obese.

**Methods:**

A mail survey collected the following data from adults living in Adelaide, Australia (n = 2210): self-reported height, weight, six LTSB, LTPA and sociodemographic variables. Participants were categorised into four groups according to their level of LTSB (dichotomised into low and high levels around the median) and LTPA (sufficient: ≥ 2.5 hr/wk; insufficient: < 2.5 hr/wk). Logistic regression analysis examined the odds of being overweight or obese (body mass index ≥ 25 kg/m^2^) by the combined categories.

**Results:**

The odds of being overweight or obese relative to the reference category (low sedentary behaviour time and sufficient physical activity) were: 1.54 (95% confidence interval [CI]: 1.20–1.98) for the combination of low sedentary behaviour time and insufficient physical activity; 1.55 (95% CI: 1.20–2.02) for the combination of high sedentary behaviour time and sufficient physical activity; and 2.26 (95% CI: 1.75–2.92) for the combination of high sedentary behaviour time and insufficient physical activity.

**Conclusion:**

Those who spent more time in sedentary behaviours (but were sufficiently physically active) and those who were insufficiently active (but spent less time in sedentary behaviour) had a similar risk of being overweight or obese. Reducing leisure-time sedentary behaviours may be as important as increasing leisure-time physical activity as a strategy to fight against obesity in adults.

## Background

Sedentary behaviours involving sitting or lying down are characterised by a low MET (metabolic equivalent) value <2 [1], and are related adversely to metabolic biomarkers and to poorer health outcomes [2,3]. Significant associations between prolonged sitting time and being overweight or obese have been reported in adults [4,5]. A population-based cohort study of Australian women found that those who gained weight during a four-year period reported spending more time sitting, compared to those who maintained their weight [6]. Specific sedentary behaviours such as television viewing [7-12] and sitting in automobiles [13,14] are known to contribute to obesity risk. Lack of moderate- or vigorous-intensity physical activity, which is identified as a MET value ≥ 3 [1], has also been associated with a higher likelihood of being overweight or obese [15,16] and weight gain [17,18].

Sedentary behaviour is not necessarily the same as a lack of physical activity [19]: an individual can meet or exceed the public health guidelines for physical activity [20], yet still spend a considerable amount of time engaged in sedentary behaviours. Conversely, those who do not meet the public health guidelines for physical activity could nevertheless engage in high volumes of light-intensity activities (with a MET value between 2 and 3), which typically include household tasks: such persons would have low volumes of sedentary time. Thus spending less time in moderate to vigorous physical activity would not necessarily lead to longer time spent in sedentary behaviour, or vice versa. This suggests that sedentary behaviour and moderate to vigorous physical activity can be independent from each other and coexist, which is supported by studies that show weak correlations between the two behaviours [7,9-11,21].

Given their independent associations with metabolic health, and their behavioural independence, it is of interest to examine how sedentary behaviours might influence obesity in the presence (or in the absence) of physical activity. Previous studies have shown that high television viewing time is associated with an increased likelihood of obesity, independent of participation in leisure-time physical activity [7-12]. However, the degree to which the combination of multiple leisure-time sedentary behaviours with physical activity may contribute to the risk of obesity is not well understood. We examined the joint associations of the total time spent in six leisure-time sedentary behaviours and physical activity with risk of being overweight or obese in a large sample of Australian adults.

## Methods

### Participants

This observational study was conducted in Adelaide, Australia during 2003–2004. A detailed description of data collection methods and response rates has been described elsewhere [22]. Briefly, the study sample was drawn from residential addresses within 32 urban and suburban neighbourhoods in the city of Adelaide. In each neighbourhood, 250 addresses were randomly selected and residents aged between 20 and 65 years were invited to participate. Eligible respondents who agreed to participate were mailed a survey. The number of responses was 2650. The return rate for those who completed the survey, calculated as a proportion of those known to be contacted, was 74.2%. The Behavioural and Social Sciences Ethics Committee of the University of Queensland approved the study.

### Measures

#### Outcome variable

The outcome measure of this study was based on body mass index (BMI), computed from participants' self-reported height and weight. It was dichotomised as either normal weight (< 25 kg/m^2^), or as overweight or obese (≥ 25 kg/m^2^) for regression analysis.

#### Leisure-time sedentary behaviours and physical activity

Participants reported the duration of the following seven leisure-time sedentary behaviours (LTSB) undertaken in the past seven days: television or video watching; computer and internet use for leisure; video game use; reading; sitting and talking with friends or listening to music; talking on the telephone; and driving or riding in a car for leisure [23]. These behaviours were chosen from the typical leisure-time sedentary behaviours identified in Australian time-use studies [24]. This sedentary behaviour instrument has previously been shown to have acceptable reliability and validity. The test-retest reliability of the items was found to be moderate to high, ranging between 0.6 and 0.8, except for listening to music (0.37) and for talking on the telephone (0.06) [23]. Validity (examined as correlations with three-day behavioural log data) was significant but moderate, ranging from 0.2 for reading to 0.6 for computer use. Talking on the telephone was excluded from analysis due to its low reliability. The total time spent in the six remaining LTSB was dichotomised into low and high levels around the median (206 min/day). Leisure-time physical activity (LTPA) was assessed with the International Physical Activity Questionnaire [25]. Participants were asked to recall the frequency and average duration of leisure-time vigorous-intensity, moderate-intensity and walking activities in the last seven days. The total amount of LTPA was classified into sufficient (≥ 2.5 hr/wk) and insufficient (< 2.5 hr/wk) according to the physical activity guidelines for health benefits [20]. Participants were categorised into the following four groups: Low LTSB/Sufficient LTPA (reference category); Low LTSB/Insufficient LTPA; High LTSB/Sufficient LTPA; and High LTSB/Insufficient LTPA.

#### Sociodemographic attributes

The questionnaire collected information on age, gender, educational attainment (with or without university education), work status (working or not) and household income.

### Statistical analysis

Univariate ANOVA and χ^2 ^analyses were used to identify sample characteristics, time spent in LTSB and LTPA, BMI and the proportions of those who were overweight or obese for each of the combined categories of LTSB and LTPA. Logistic regression analyses examined the odds of being overweight or obese by the combined categories of LTSB and LTPA, controlling for the sociodemographic variables. The same logistic regression analyses were carried out in men and women separately. We also examined the odds of being obese (BMI ≥ 30 kg/m^2^) compared to normal weight across the four categories for the whole sample. Analyses were conducted using SPSS version 15.0.

## Results

The final sample size was 2210 (798 men, 1412 women) after excluding participants with missing values (casewise, n = 408) and those with extreme values in BMI (< 15 kg/m^2 ^and > 50 kg/m^2^, n = 23) and in total time spent in LTSB and LTPA (> 960 min/day, n = 9). Men and women were differently distributed in the weight categories: some 60% of men (n = 470) and 45% of women (n = 633) were classified as overweight or obese (*p *< 0.001).

Table 1 shows the characteristics of the sample, according to the combined categories of LTSB and LTPA. The combined category produced evenly-divided groups, although a slightly smaller percentage of participants (22%) belonged to the High LTSB/Sufficient LTPA category. When stratified by gender, a larger proportion of men belonged to the High LTSB/Sufficient LTPA category in comparison to the other categories. The study sample underrepresented men, younger people, and those without tertiary education.

**Table 1:** Sample characteristics by the combined categories of leisure-time sedentary behaviours (LTSB) and leisure-time physical activity (LTPA)

		Combined Categories of LTSB and LTPA				
			
	Total	Low LTSB/ Sufficient LTPA	Low LTSB/ Insufficient LTPA	High LTSB/ Sufficient LTPA	High LTSB/ Insufficient LTPA	*p*
Number (%)	2210	562 (25%)	551 (25%)	491 (22%)	606 (27%)	-
Gender, % men	36.1%	34.0%	32.7%	43.6%	35.1%	< 0.01
Mean age, years (sd)	44.2 (12.3)	42.3 (12.0)	43.4 (11.5)	45.6 (13.0)	45.6 (12.3)	< 0.001
Education, % with tertiary education	49.0%	62.6%	46.2%	52.2%	36.3%	< 0.001
Work status, % working	67.5%	77.7%	73.4%	61.5%	57.5%	< 0.001
Income, % >$41,600 per annum	52.2%	66.8%	54.8%	51.3%	37.2%	< 0.001
Mean BMI, kg/m^2 ^(sd)	25.9 (5.0)	24.5 (4.2)	25.8 (4.9)	25.8 (4.8)	27.2 (5.7)	< 0.001
Weight status, % overweight or obese	49.9%	37.2%	49.9%	50.7%	61.1%	< 0.001

Table 1 also shows that participants' BMI varied with the combined categories of LTSB and LTPA. Post-hoc comparisons revealed that two extreme categories, Low LTSB/Sufficient LTPA and High LTSB/Insufficient LTPA, were significantly different in BMI from the other categories (*p *< 0.001). However, the Low LTSB/Insufficient LTPA and High LTSB/Sufficient LTPA categories were not different in their BMI values. The similar pattern was observed for the proportion of overweight or obese respondents across these categories. All the sociodemographic variables were associated with the categories of LTSB and LTPA (*p *< 0.001), with the greatest differences typically observed between the two extreme groups.

Table 2 shows the mean time reported for LTSB and LTPA, plus the contribution of each sedentary behaviour to the total LTSB. Bivariate correlation coefficients between the total time spent in LTSB and in LTPA was low (*r *= -0.07). TV viewing time occupied the largest proportion, which reached almost half of the total sedentary behaviour time in the High LTSB/Insufficient LTPA category. Table 3 shows the odds of being overweight or obese by the combined categories of LTSB and LTPA, for the whole sample, and for men and women, controlling for the sociodemographic variables. In all the analyses (the whole sample, men, women), those in the Low LTSB/Insufficient LTPA category and the high LTSB/Sufficient LTPA category had about 50% higher odds of being overweight or obese, whereas those with High LTSB/Insufficient LTPA had more than twice the odds of being overweight or obese, compared to those in the Low LTSB/Sufficient LTPA category.

**Table 2:** Mean time spent in LTSB and in LTPA and the contribution of each sedentary behaviour to the total LTSB by the combined categories of LTSB and LTPA

		Combined Categories of LTSB and LTPA				
			
	Total	Low LTSB/ Sufficient LTPA	Low LTSB/ Insufficient LTPA	High LTSB/ Sufficient LTPA	High LTSB/ Insufficient LTPA	*p*
Mean time spent in LTSB, min/day (sd)	235.1 (139.0)	134.9 (48.1)	133.5 (48.1)	322.8 (104.1)	349.2 (139.6)	< 0.001
% TV viewing	45.5%	41.6%	44.7%	46.0%	49.4%	< 0.001
% Computer and Internet use	7.1%	5.8%	6.9%	7.3%	8.3%	< 0.01
% Video game	0.7%	0.5%	0.5%	1.1%	0.8%	< 0.05
% Reading	15.1%	17.4%	15.9%	15.0%	12.4%	< 0.001
% Sitting and talking	15.6%	17.7%	14.8%	15.6%	14.4%	< 0.001
% Driving for leisure	16.0%	17.1%	17.2%	15.0%	14.7%	< 0.01
Mean time spent in LTPA, min/day (sd)	37.1 (54.4)	70.9 (60.8)	5.8 (6.8)	73.1 (63.6)	5.1 (6.6)	< 0.001

**Table 3:** Odds (95% confidence intervals) of being overweight or obese according to the combined categories of LTSB and LTPA

LTSB/LTPA category	Total (n = 2116)	Men (n = 770)	Women (n = 1346)
Low LTSB/Sufficient LTPA	1.00	1.00	1.00
Low LTSB/Insufficient LTPA	1.54 (1.20–1.98)***	1.49 (0.97–2.29)	1.57 (1.15–2.15)**
High LTSB/Sufficient LTPA	1.55 (1.20–2.02)***	1.43 (0.95–2.16)	1.64 (1.16–2.31)**
High LTSB/Insufficient LTPA	2.26 (1.75–2.92)***	2.21 (1.43–3.40)***	2.28 (1.66–3.13)***

** *p *< 0.01, *** *p *< 0.001

Adjusted for gender (whole sample), age, education, work status, and household income

When the distinct odds of being obese (compared to normal weight) was examined across the four categories, those in the Low LTSB/Insufficient LTPA category (OR = 1.87, 95% CI: 1.26–2.75) and the high LTSB/Sufficient LTPA category (OR = 1.96, 95% CI: 1.31–2.92) again had similar odds for being obese; while those in the High LTSB/Insufficient LTPA category had over 3.5 times the odds of being obese (OR = 3.70, 95% CI: 2.55–5.37), compared to the Low LTSB/Sufficient LTPA category.

## Discussion

This study examined the joint associations of total time spent in six leisure-time sedentary behaviours, and in leisure-time physical activity, with the odds of being overweight or obese. As expected, those who spent more time in sedentary behaviours and were not sufficiently physically active had an increased likelihood of being overweight or obese: the odds of being overweight or obese were 2.3 times higher and the odds of being obese were 3.7 times higher compared to those with low sedentary behaviour time and sufficient physical activity. The combination of less time in sedentary behaviours and insufficient physical activity and that of more sedentary behaviour time and sufficient physical activity were similarly associated with the risk of being overweight or obese. In the analysis conducted on the whole sample (men and women combined), these two groups had more than 50% higher odds of overweight or obesity and almost twice the odds of obesity, compared to those who had less sedentary behaviour time and were sufficiently physically active. It can be argued that reduced energy expenditure from lack of light-, moderate- or vigorous-intensity physical activity or increased energy intake which could occur while being engaged in sedentary behaviours (or both) may be responsible for the increased likelihood of overweight and obesity observed in this study.

These findings suggest that high levels of overall sedentary behaviour time may contribute to obesity potentially as much as does lack of moderate to vigorous physical activity. They also suggest that even if adults meet the public health guideline for leisure-time physical activity [20], they may have a high risk of being overweight or obese if they spend a large amount of time in sedentary behaviours during leisure. Our findings are consistent with past studies that showed the associations of a particular sedentary behaviour (TV viewing) with weight status independent of physical activity levels [7-12]. A weak relationship between sedentary behaviours and moderate to vigorous physical activity, which has been reported in previous studies [7,9-11,21], was also confirmed in this study.

It was found that participants on average reported about 4 hours on LTSB per day. This is comparable with findings obtained from Dutch workers (4.7 hours/day) [26], and from middle-aged French adults (3.4 hours/day, which included only TV watching, computer use and reading) [27]. TV viewing was the largest component, occupying 45% of the total LTSB that was assessed. Among those who were categorised as having a high volume of LTSB, TV viewing time occupied an even larger proportion, which suggests that prolonged TV viewing time may contribute to a higher volume of total sedentary behaviour time.

Men and women differed in the proportions of those who were overweight or obese, and in the proportions of membership to the combined LTSB/LTPA categories: more men belonged to the overweight or obese category, and to the category of high LTSB and sufficient LTPA. Despite these differences, regression analyses found a similar pattern of associations between the combined categories and being overweight or obese in men and women, although the odds for the combination of low LTSB and insufficient LTPA and that of high LTSB and sufficient LTPA were not significant in men, potentially due to a smaller sample size. Thus, both in men and in women, the findings suggest that the risk of overweight or obesity associated with more sedentary leisure time is as high as not being sufficiently physically active during leisure time. However, past studies have shown stronger associations of sedentary behaviour with metabolic health risks in women [7,8,27,28]. Given the gender imbalance in our study sample, we are reluctant to place an emphasis on any apparent gender differences in the findings. Further research should investigate whether and to what extent gender may moderate the relationship between sedentary behaviours and health indictors, and the potential mechanisms (behavioural or biological) that may underlie the differences between men and women.

We found that socio-economic (education and income) and work status were related to sedentary behaviour and physical activity. High levels of LTSB appeared to be more common among those in lower socio-economic groups and in those without work (who may have more discretionary time available to them). A study in The Netherlands found an association between physical inactivity and neighbourhood social and environmental inequalities [29]. Although physical activity and sedentary behaviour are not closely correlated, it may be hypothesised that less opportunities for social and recreational activity in neighbourhoods may have a bearing on the time spent being sedentary. Indeed, a recent study has shown that women living in "walkable" neighbourhoods tend to spend less time in TV viewing, compared to those living in less-walkable areas [30]. Further research is needed to examine how social and environmental factors may be related to prolonged sedentary behaviour time.

Methodological limitations include the cross-sectional nature of the study, which precludes causal inferences, and self-report measures of weight, height, sedentary behaviours and physical activity. Longitudinal studies comparing the effects of reducing sedentary behaviours and increasing physical activity are needed to examine the possible causal nature of the relationships that we have identified. Also as stated before, the low proportion of men in our sample limits our ability to examine the gender differences that have emerged in previous studies.

This study adds to a growing body of evidence supporting the potential health benefits of reducing sedentary behaviour time. Sedentary behaviours should not be considered as simply being the bottom end of the physical activity continuum, and should be addressed specifically and explicitly, with attention to their distinct health impacts and determinants [23,31,32]. Our findings suggest the need for a stronger focus on reducing time spent in leisure-time sedentary behaviours to decrease the risk of obesity and associated chronic diseases.

## List of Abbreviations

BMI: Body mass index; LTPA: Leisure-time physical activity; LTSB: Leisure-time sedentary behaviour.

## Competing interests

The authors declare that they have no competing interests.

## Authors' contributions

TS conceived the idea, analysed the data and drafted the manuscript. GH, DD, JS and NO contributed to the writing and assisted with the analysis and interpretation. NO is the principal investigator of the project. All authors have read and approved the final manuscript.
